# Analysis of EDM Performance, through a Thermal–Electrical Model with a Trunk-Conical Discharge Channel, Using a Steel Tool and an Aluminium Workpiece

**DOI:** 10.3390/ma14113038

**Published:** 2021-06-03

**Authors:** José A. S. Almacinha, Alice M. G. Fernandes, Duarte A. Maciel, Ricardo J. M. Seca, José D. R. Marafona

**Affiliations:** Departamento de Engenharia Mecânica, Faculdade de Engenharia da Universidade do Porto, Rua Roberto Frias, 4200-465 Porto, Portugal; jasa@fe.up.pt (J.A.S.A.); up201606564@fe.up.pt (A.M.G.F.); up201605880@fe.up.pt (D.A.M.); up201603630@fe.up.pt (R.J.M.S.)

**Keywords:** EDM, trunk-conical discharge channel, steel, aluminium, FEM, tool wear rate, material removal rate, tool wear ratio, workpiece surface roughness

## Abstract

In this article, a finite element (FE) thermal–electrical model with a trunk-conical discharge channel is employed to simulate individual EDM discharges with a time-on of 18 μs up to 320 μs, which are subsequently compared with the experimental results to validate the model. The discharge channel is a trunk-conical electrical conductor which dissipates heat by the Joule heating effect, being the correspondent factor equal to 1. Instead of the usual copper–iron electrode combination, steel (DIN CK45) and aluminium alloys (DIN 3.4365) are the implemented materials on both the tool and the workpiece, respectively. The numerical results were measured using the melting temperature of the materials as the boundary of material removal. The results obtained with the thermal–electrical model, namely the tool wear ratio, the tool wear rate, the material removal rate, and the surface roughness, are in good agreement with experimental results, showing that the new FE model is capable of predicting accurately with different materials for the electrodes.

## 1. Introduction

Electrical discharge machining (EDM) is a thermal–electrical manufacturing process that employs the subtracting ability of electrical discharges in the removal of conductive material. In this method, the tool and the workpiece are made electrodes and placed in a suitable electrical circuit. Both the workpiece and the tool must be emerged in a dielectric fluid in order to concentrate the discharge energy, to clean the particles removed from the electrodes and to avoid their electrolysis.

EDM was first discovered by the Soviet brothers Lazarenko during several experiments about the wear of materials under an electrical current, as they were trying to find the best combination of metals and alloys to resist electrical arches [1].

Since its finding in the 1940s, this technology has been in constant development towards the industrial production area, being crucial in the manufacturing of moulds and tools, prototyping, texturing, and everything that requires micrometric, complex and precise machining [1].

This machining process is not limited by the material hardness, as long as the material is electrically conductive, which makes this manufacturing method more appealing than most of the conventional ones. Since there is no direct contact between the tool and the workpiece, there is no mechanical stress involved, which suppresses the need for complex blockage systems and avoids wear due to friction [2].

Due to its intrinsic complexity and uncertainty, several models by various authors have been constructed over time to simulate the EDM process. These can be divided in the following categories: thermal, thermal–electrical, and thermal–hydraulic.

The electrical discharge machining modelling resorts to a series of distinct models. Amongst these, there is a large number of thermal models [3,4,5,6], very few thermal–electrical models [7,8] and only one thermal–hydraulic model [9]. As far as thermal models are concerned, different sources of heat are used, such as the point heat source, the disc heat source, and Gaussian heat distribution source. K. Salonitis et al. [10] proposed a thermal model in which they consider the EDM process as a one-dimensional conduction problem. However, they analyse only the workpiece behaviour. Therefore, the authors only take into account the thermal properties of the workpiece’s material. The point heat thermal model [5] has been applied to anode (−), where it is considered that the heat source is near the anode (−) and far from the cathode (+). So, the disc heat source is applied to cathode (+) because it is far from the heat source [6]. The Gaussian heat distribution source is applied to the anode (−) and in the research done [4], one Gaussian heat distribution source affects another Gaussian heat distribution by its heat effect. Moreover, these models do not take into account the relationship between the gap of the electrodes (*G*) and the applied voltage (*E*). This gap is maintained stable during the machining, through adaptive control (AC), regardless of the changing conditions that may arise. Therefore, the thermal–electrical models take into account the gap of the electrodes (*G*) and its relationship with applied voltage (*E*) or discharge current (*I*). Eubank, P.T. et al. [11] defined the relationship between the gap of the electrodes (*G* [μm]) and the discharge current (*I* [A]) through Equation (1).

(1) G=10.268+8.984×I

The equivalent radius of the discharge channel is shown in research [12] through the Equation (2) as function of discharge current (*I*) and time-on ($t_{on}$).

(2) $$r=2.04\times 10^{-3}\times I^{0.43}\times t_{on}^{0.44}$$

All these variables are related with the electrical conductivity (Ω) of the dielectric in Equation (3) that was used in the research done [8].

(3) $$\Omega =\frac{G\times I}{S\times E}$$

where *G* (cm) is the gap width, *I* (A) is the current intensity in the discharge channel, *S* (cm${}^{2}$) is the discharge channel section area and *E* (V) is the work voltage. The discharge channel radius is calculated using the circular area formula [7,8].

Graphite and copper are the most used electrode materials. These tool materials achieve large and medium material removal rate, respectively, with minimum tool wear rate. The relationships between EDM output parameters and the EDM input parameters, such as the discharge current (*I*), the gap voltage (*E*), the time-on ($t_{on}$), the time-off ($t_{off}$), etc., were studied in [13,14,15]. Material removal rate had an accelerated increase through an increase in the discharge current and in the applied voltage, but a decelerated increase with time-on ($t_{on}$), in the research [16].

Therefore, from this literature review about EDM modelling, one can conclude that the researches [5,6] used different heat sources, a disc heat source for cathode (+) and a point heat source for anode (−). However, it was not implemented in an integral thermal model. Thus, the present research proposes and implements an thermal–electrical model based on the philosophy of point–disc heat source applied to anode (−) and cathode (+), respectively, or by other words, a trunk-conical discharge channel, which leads the thermal–electrical heat source closer to the anode (−) and farther from the cathode (+). Moreover, the combination of both electrodes materials, tool and workpiece, used in EDM modelling, is copper and mild steel, although the present research did the EDM performance analysis with the combination of a tool in mild steel and a workpiece in aluminium 7075 to find the possibility to use a cheaper tool in a workpiece material applied in aerospace structures. This combination of tool and workpiece materials was studied for the first time in the present research.

### 1.1. EDM Process

EDM begins by obtaining a certain electric potential gradient between both electrodes, creating an electrical field in the gap. Then, the dielectric fluid in the gap starts ionising, as the cathode releases electrons, and it becomes conductive. The voltage stabilises, after the ignition phase ends, allowing the current to flow in the circuit [3,17].

**Figure 1:**
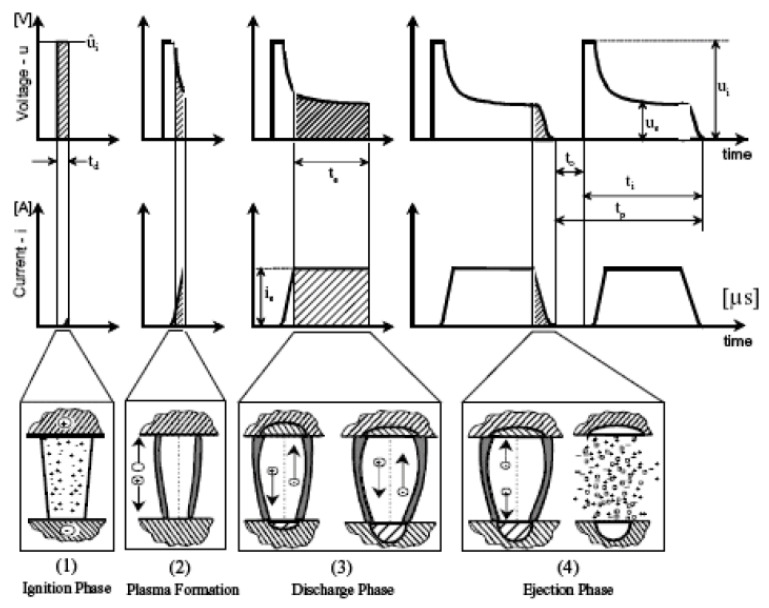
EDM phases [18].

The fluid, in a plasma state, promotes the melting and evaporation of the electrode material through the kinetic energy of its particles. A complementary shape of the tool is then printed in the workpiece. Multiple consecutive discharges result in several craters that will then create the intended outcome [3]. The main stages of the process are illustrated in Figure 1.

#### 1.1.1. Ignition Phase

In the first stage, namely the ignition phase, the tool approaches the workpiece until the gap is shortened to a length capable of allowing the current flow. At that point, a voltage differential, high enough to break the dielectric strength of the gap fluid, is then applied between both electrodes. The ignition delay represents the time between the voltage pulse generation and the start of the current flow. It is needed so that the initial electrons react with the neutral environment of the dielectric fluid, forming a bridge between the two electrodes. During the plasma formation, the voltage drops while the current increases, leading to the second phase [17].

#### 1.1.2. Discharge Phase

When the plasma becomes sufficiently conductive, both the voltage and the current stabilise and an electrical discharge occurs. During this phase, the temperature can rise up to 12,000 K, fusing the material and promoting its removal from both electrodes.

#### 1.1.3. Ejection Phase

After the discharge, both the voltage and current drop, decreasing the temperature. The dielectric liquid cools the melted and evaporated materials which solidify to form spherical debris particles. Thus, debris particles are removed from the gap with the dielectric liquid to avoid their possible reattachment to the electrode surfaces.

#### 1.1.4. Time-Off

When the electrical pulse ends, it is fundamental to have a small interval of time before the next one begins. During this time, the gap fluid regains its non-conductive properties, the system cools down, the debris is ejected, and the tool is re-positioned. This interval is important in order to preserve the machining conditions after each pulse [17,19].

#### 1.1.5. Electrode Performance

In order to evaluate an EDM process and its efficiency, the material removal rate (MRR), the tool wear rate (TW), the tool wear ratio (TWR) and the workpiece’s surface roughness must be measured. As expected, a lower TWR translates into more efficient process regarding the removal of material from the workpiece, even though its surface roughness has an influence on the overall efficiency of the EDM process and must be considered.

Each of these material removal parameters are influenced differently by time-on, time-off, applied voltage and current, electrode materials and their physical properties, gap, and dielectric fluid.

An increase in the discharge duration causes an increase in the material removal rate until it reaches a limit, after which any further increase leads to a decrease in the workpiece’s MRR. As the discharge duration gets longer, the energy effects are experienced for a longer period of time, making debris clearing and restoration of the dielectric fluid properties much more difficult [20].

The tool wear ratio decreases with the increase in time-on.

With a discharge duration increase, the energy involved also increases, resulting in a larger degradation and dispersion of craters on the workpiece’s surface, which implies a worse surface roughness [21].

#### 1.1.6. Metallurgical Effects

The machined surface is affected by the heat and the chemical and physical changes inherent to the process. These occurrences modify the material properties and influence the integrity of the final product in several layers.

The first layer appears due to the material deposition of solidified debris onto the workpiece surface and is easily removed by grinding. The second layer is called the recast layer and appears due to the high temperatures involved in the process. The chemical reactions that take place in the gap and in the electrodes form some elements which migrate to the workpiece.

The third layer is called the heat affected zone (HAZ) and it is the layer of material that was heated, changing its material properties. Nonetheless, the material itself is not removed [22].

Sometimes, deposition of carbon or other elements derived from the dielectric fluid and other chemical reactions produce a “black layer” on the tool surface. This has an influence in the electrode consumption and consequently in the tool wear [23].

## 2. Materials and Methods

### 2.1. Research Goals

For this article, the authors established a series of objectives. Performing the EDM process in the laboratory to confirm the numerical results using an innovative pair of materials, namely steel and aluminium as electrodes of the circuit, was the first step. This is where the innovative part of the experimental research done by the authors is focused on.

Secondly, simulations of the EDM process using Abaqus/Standard software through a thermal–electrical model that uses a trunk-conical discharge channel as the main new part of this model were ran. Eubank et al. [11] made mention of this fact, stating that it is very difficult to implement it in a numerical simulation. The other common point to the experimental runs are the steel and aluminium electrodes of the electrical circuit. Research shows that it is possible to machine an aluminium workpiece with a steel tool with reasonable wear of the tool.

Finally, the authors compared both results. The comparison of both results will show that, with the present thermal-electrical model, it is possible to achieve the better tool material for a determined workpiece material. Therefore, the aluminium 7075 was chosen because it is widely used in aerospace structures due to its high strength and low density. Moreover, the graphite and copper like the mild steel (CK45) is a material of the electrode capable of being manufactured by conventional machines, allowing low machining costs.

### 2.2. Process Modelling

The model adopted in this finite element analysis (FEA) is based on the thermal–electrical one by [8], that is possible to be carried on by the Abaqus/Standard software, since it provides a thermal–electrical coupling to analyse this type of problems. The thermal–electrical elements used incorporate both temperature and electrical potential as nodal variables. Several heat sources have been analysed over the years, being the main ones the Gaussian, the point and the disc heat sources. These sources are applied on the surface of the workpiece and tool, which are in contact with the discharge channel.

For this report, some assumptions derived from the mentioned EDM model were made. Firstly, the domain is considered axisymmetric and the outer cylinder is adiabatic. Secondly, the discharge channel has a truncated cone shape. Its radii will be calculated later in this paper. Additionally, it was assumed that there is a conversion of the electrical energy into thermal energy—Joule heating—in the discharge channel. However, for purposes of better approximation to the experimental results, the Joule heating factor will be considered as 1. Additionally, the workpiece and the tool are homogeneous and isotropic and most material properties of the electrodes and dielectric are temperature-independent, apart from the thermal conductivity.

Moreover, it is important to mention that the heat transfer to the electrodes occurs by conduction. The model includes the heat exchange by radiation between the discharge channel and the surrounding dielectric, which is considered at the ambient temperature, namely 288 K. The forced heat convection caused by the fluid flowing through the mesh is not considered. In addition, the analysis is done for a single discharge, considering the reduction in time-on due to multiple discharges and the electrical conductivity is independent of the electrical field and temperature. Finally, the relationship between the gap of the electrodes (*G* (μm)) and the applied voltage (*E*) is described in Equation (4)

(4) G=27.78×E

#### Governing Equation

The governing equation for the coupled thermal—electrical problem [24] can be derived from the Maxwell’s equation of conservation of charge in a conducting material. The conservation of charge means that electrical charges cannot be created or destroyed. Assuming a steady-state direct current, the equation reduces to

(5) $$\int_{S}^{}J\times n\;dS=\int_{V}^{}r_{c}\;dV$$

where *V* is a control volume with a surface *S*, *n* is the outward normal to *S*, *J* is the electrical current density, which is the current per unit of area, and $r_{c}$ is the internal volumetric current source per unit of volume. Using the divergence theorem, one can convert the surface integral into a volume integral written in the differential form

(6) $$\int_{V}^{}\left[\frac{\partial }{\partial x}J-r_{c}\right]\;dV=0$$

and since the volume is arbitrary, this provides the point wise differential equation

(7) $$\frac{\partial }{\partial x}J-r_{c}=0$$

The equivalent weak form is obtained by introducing an arbitrary variational, the electric potential field, δφ, and integrating over the volume. As the total number of discharges that originates the gap is not known and the variability of the gap after the different positions that the ram takes during the erosion process is extremely hard to control, the volume cannot be calculated with the highest precision:

(8) $$\int_{V}^{}\delta \varphi \left[\frac{\partial }{\partial x}-J-r_{c}\right]\;dV=0$$

Using the first chain rule and then the divergence theorem, this statement can be rewritten as

(9) $$-\int_{v}^{}\frac{\partial \delta \varphi }{\partial x}J\;dV=\int_{S}^{}\partial \varphi J\;dS+\int_{V}^{}\partial \varphi r_{c}\;dV$$

where *J*, defined as −J×n, is the current density entering the control volume across *S*. Equation (9) is the governing equation of the coupled thermal-electrical problem.

### 2.3. Constitutive Behaviour

The flow of the electrical current can be described by Ohm’s Law

(10) $$J=\sigma^{E}\times E$$

where $\sigma^{E}$ (θ, $f^{\alpha }$) is the electrical conductivity matrix; θ is the temperature; and $f^{\alpha }$, α=1,2,…, are any predefined field variables. The conductivity can be isotropic, orthotropic, or fully anisotropic, but in this paper, the isotropic case is considered. E(x) is the electric field intensity defined as

(11) $$E=-\frac{\partial \varphi }{\partial x}$$

Introducing the Ohm’s Law, the governing equation of the conservation of charge becomes

(12) $$\int_{V}^{}\frac{\partial \delta \varphi }{\partial x}\times \sigma^{E}\times \frac{\partial \varphi }{\partial x}\;dV=\int_{S}^{}\delta \varphi J\;dS+\int_{V}^{}\delta \varphi r_{c}\;dV$$

#### Thermal Energy Balance and Surface Conditions

The thermal energy balance and surface conditions are presented in detail in references [7,24].

Considering the surface interaction effects and the electric energy released as thermal energy, the governing electric, and thermal equations become

(13) $$\int_{V}^{}\frac{\partial \delta \varphi }{\partial x}\times \sigma^{E}\times \frac{\partial \varphi }{\partial x}\;dV=\int_{V}^{}\delta \varphi r_{c}dV+\int_{S_{p}}^{}\delta \varphi JdS+\int_{S_{i}}^{}\delta \varphi \sigma_{g}\left(\varphi_{B}-\varphi \right)\;dS$$

and

(14) $$\begin{array}{c} \int_{V}^{}\rho \dot{U}\delta \theta dV+\int_{V}^{}\frac{\partial \delta \theta }{\partial x}\times k\times \frac{\partial \theta }{\partial x}dV=\\ \;=\int_{V}^{}\delta \theta rdV+\int_{V}\delta \theta \eta_{g}P_{ec}dV+\int_{S_{p}}^{}\delta \theta qdS+\\ \;+\int_{S_{i}}\delta \theta \left(q_{c}+q_{r}+q_{ec}\right)dS \end{array}$$

In the finite element method (FEM), the equilibrium is approximated as a finite set of equations by introducing interpolation functions. The discretised quantities represent nodal variables, with nodes shared between adjacent elements. An appropriate interpolation is chosen to provide adequate continuity of the assumed variation. There is an adequate interpolating function for the virtual electric potential and the temperature fields in the thermal problem, transforming Equations (13) and (14) into a set of discretised electric and thermal expressions.

The governing equations of the coupled thermal—electrical problem and of the charge equation for the constitutive behaviour and their deductions are presented in the referenced literature [7] but will not be discussed any further in this paper. Furthermore, the same applies to some other mathematical formulations, like the thermal energy due to electrical current, the surface conditions, the spatial discretisation, the discharge channel radius and the heat flux and energy–power partition.

### 2.4. Assumptions

#### 2.4.1. Discharge and Plasma Channels

The discharge channel is a result of the dielectric oil breakdown, and it is bound to have constant plasma-like properties throughout the entirety of the discharges. This consistency is due to the stabilisation after the dielectric breakdown of temperature and pressure inside, the latter reaching its maximum value immediately after the dielectric breakdown, without suffering major variations during the electrical discharges. This channel conducts the electrical current due to its level of ionisation [17], so that the values of this variable and the respective value of vaporisation stay constant throughout all of the discharges. The radius of the discharge channel is heavily influenced by the current of the discharge and by the small time period that is needed for the dielectric breakdown (ignition delay). The control of the voltage values will lead to an increase in the discharge channel width. Therefore, due to this variation of width, the maximum temperature point on the discharge channel will move closer to the segment with higher current density, increasing the probability of a following electrical discharge to occur. Consequently, the radius of the discharge channel is governed by a linear function, resulting in a trunk-conical shape. The discharge gap is kept constant throughout the whole discharge by a servo control system.

The thermal energy originated by the current intensity flux will be considered as the heat source for both electrodes, according to the Joule heating effect. The electrical conductivity (Ohm · cm)${}^{-1}$ used in the model can be mathematically translated by Equation (3).

#### 2.4.2. Heat Distribution

The discharge duration, its current and the gap between the electrodes, or work voltage, are all variables that have an impact on the energy used to remove material, as it is possible to see it in the following equation:

(15) $$W=U_{e}\times I_{e}\times t_{e}$$

Even though only a fraction of the discharge energy is used in a single discharge, it is assumed that the entirety of the energy spent on the discharge pulse can be used for multiple discharges. This increases the material removal rate per pulse time and causes the temperature on the channel to drop down [25].

#### 2.4.3. Volume of Erosion for a Unique Discharge or Multiple Discharges

Through the FEM analysis, the volume of the eroded material for a single discharge is calculated. The melting temperature of the materials will limit the amount of material removed from the electrodes. Additionally, and because the energy source has a circular shape, the erosion crater will be shaped similarly to a spherical dome. Consequently, the removed volume (*V*) is calculated through a relation between the radius of the molten area (*r*) and its respective depth (*h*), as it is possible to see in the following equation:

(16) $$Volume=\frac{\pi }{6}\times h\times \left(3r^{2}+h^{2}\right)$$

The material removal rate is obtained by dividing the removed volume of the workpiece’s material by the sum of the time-on and time-off values. The tool wear rate is calculated using the exact same method applied to the volume of used tool’s material. Additionally, the tool wear ratio is determined by the ratio between the tool and workpiece’s removed volumes. Finally, the depth of the crater of removed material is specified in micrometers and it stands for the maximum height of the workpiece’s surface roughness profile.

The values of TW are related to the increase in the work gap applied. Basically, the workpiece’s removed material allows for a relative receding movement of the electrode tool, because this point is distant from the maximum temperature point on the discharge channel.

#### 2.4.4. Assumed Properties

The FEM simulations were done using the software Abaqus, which allows a fairly quick and steady workflow, since thermal–electric elements can be used to model the process. An axisymmetric approximation was used to simplify the problem at hand.

**Figure 2:**
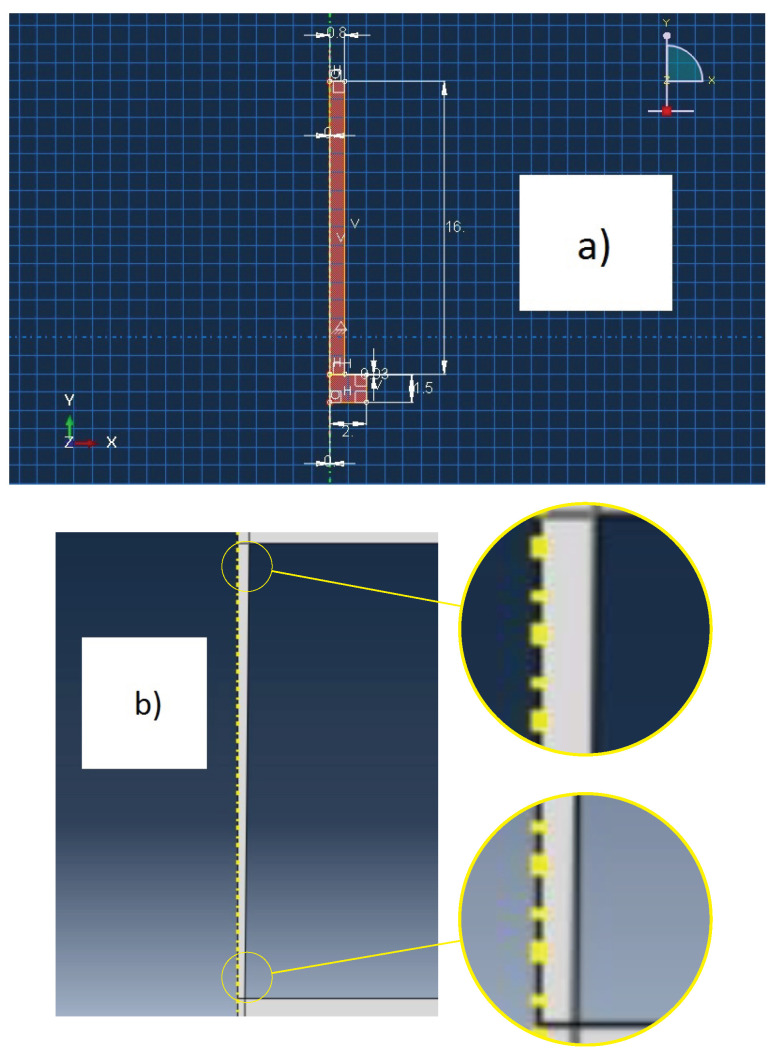
(**a**) Model sketch; (**b**) Shape of the gap.

The dimensions of the electrode are 16 cm and 0.8 cm of length and radius, respectively, while the workpiece has a radius of 2 cm and a height of 1.5 cm. A gap of 0.03 cm was used, calculated with the help of Equation (4). In order to calculate the radius of the discharge channel, Equation (3) was employed, using Ω = 75,000 (Ohm · cm)${}^{-1}$, *E* = 15 V, and *I* = 19.3 A, for a time-on of 18 μs. The sketch which defines the geometry of the model and the shape of the gap can be seen in Figure 2a,b. Despite the experimental tool being square, a circular bar with the same area was used in the simulation, meaning that the current density ends up being similar. In the latter, the trunk-conical shape is formed due to the high travel speed of the electrons from the workpiece to the tool, resulting in an accumulation of electrons around the tool’s lower border. On the other hand, due to slower travel speed of the protons from the tool to the workpiece, the accumulation of protons is less intense around the workpiece’s upper border.

The most relevant material properties for this model had to be specified in Table 1.

**Figure 3:**
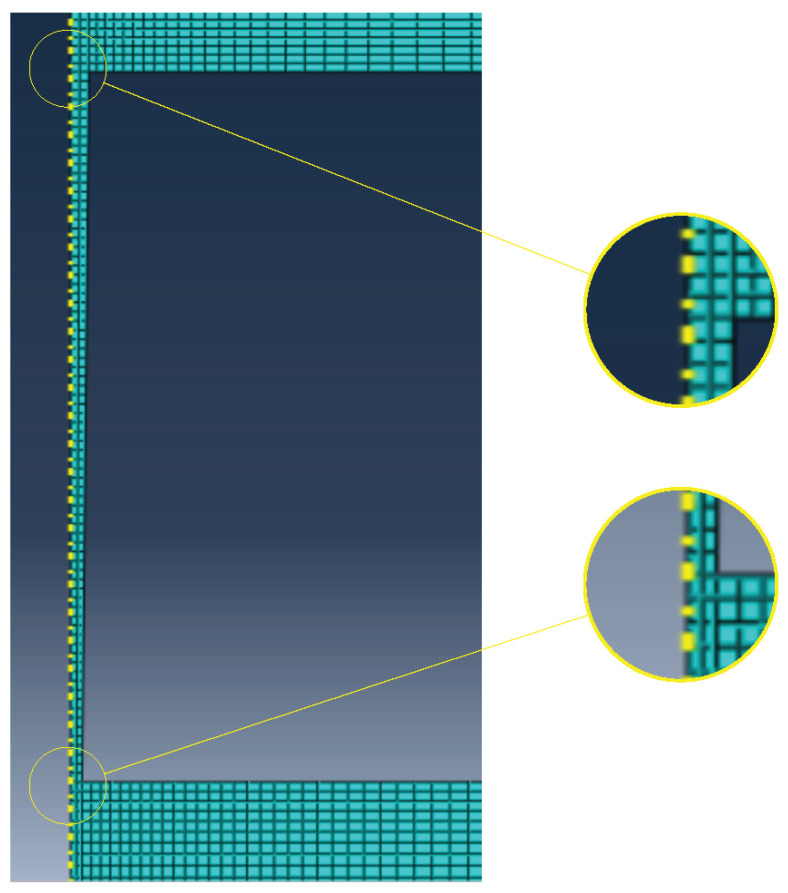
Mesh biased towards the channel.

The finite element mesh presents a refined discharge channel and its neighbours in order to better reproduce the high discharge channel temperature variation. The mesh used can be seen in Figure 3.

The finite elements used for this simulation are thermal–electrical elements with the designation DCAX4E, available in Abaqus software. 165,000 elements were used to obtain the numerical results.

Before the simulation could start, further general and boundary conditions were defined, for the time-on of 18 μs, such as the initial temperature of the system, which was set to 288 K; the electrical potential in the tool was 15 V; the current intensity was 19.3 A and the Stefan–Boltzmann constant was taken into account.

### 2.5. Experimental Parameters and Methodology

The experimental procedures were performed in a die-sinking EDM machine AGIE COMPACT 3, from the manufacturer AGIE, Lausanne, Switzerland. It is equipped with an adaptive control optimisation (ACO) system which enables an automatic process optimisation, that was switched off in order to allow the results to be adaptable to different machines. The electrode tool and workpiece materials are DIN CK45 steel and aluminium alloy 7075-DIN 3.4365, respectively, from the supplier Thyssen, Queluz, Portugal. The process performance is evaluated using the MRR, TW and TWR metrics, which are determined by an average value of the weight variation, with each value being measured five times by a Kern PLS balance with an accuracy of 0.01 grams, from Kern, Albstadt, Germany, that is converted in volume through the density of the materials. This value is then divided by the machining time to obtain the mentioned parameters. The average maximum height of the workpiece surface roughness profile, *R*z, and the maximum height of the workpiece surface roughness profile, *R*max, were chosen to characterise the workpiece surface roughness, because the depth of the crater obtained in the numerical simulation is considered equal to the average maximum height of the workpiece surface roughness profile. The average maximum height of the workpiece surface roughness profile, *R*z, was assessed through five measurements using a Hommelwerk T4000 measurement instrument, from Hommelwerk, Villingen-Schwenningen, Germany.

The experiences were performed at the DEMec laboratory in FEUP, Porto. The parameters defined in the EDM machine are presented in Table 2.

**Figure 4:**
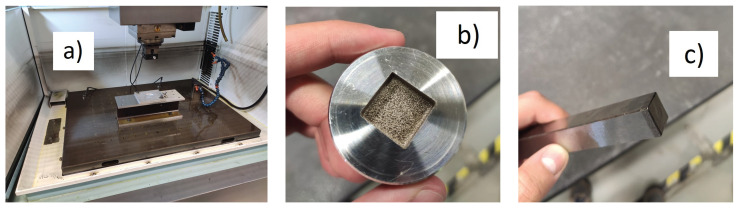
(**a**) EDM machine setup; (**b**) Workpiece; (**c**) Tool.

The dielectric fluid used in these experiments was hydrocarbon oil. The tool had a prismatic shape, while the workpiece was a cylinder. In Figure 4a–c, the EDM setup is shown, as well as the tool and workpiece after the experiment.

## 3. Results

### 3.1. Simulation Results

**Figure 5:**
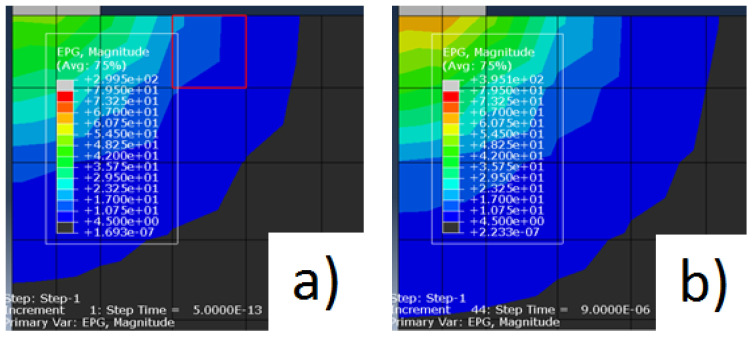
(**a**) Electrical potential gradient for the pulse of 18 μs at the beginning of the discharge; (**b**) Electrical potential gradient for the pulse of 18 μs at the end of the discharge.

**Figure 6:**
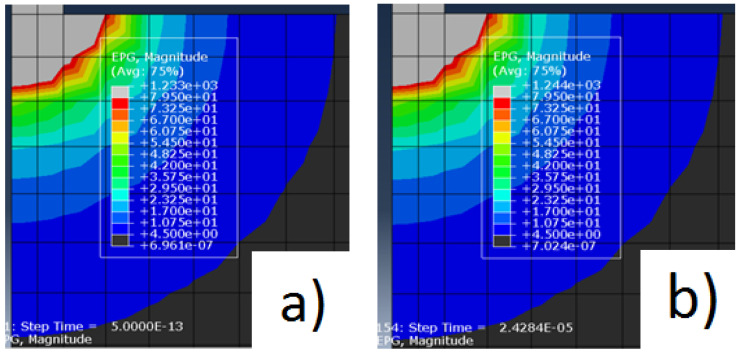
(**a**) Electrical potential gradient for the pulse of 320 μs at the beginning of the discharge; (**b**) Electrical potential gradient for the pulse of 320 μs at the end of the discharge.

Examples of some simulation results can be seen in Figure 5a,b and Figure 6a,b.

As the scale is equal between both figures of equal pulses, we can clearly notice a difference between the area of influence of the electrical potential gradient at the beginning and at the end of the discharges for both pulse values, being that at the end of the discharge, the electrical potential gradient is higher than at the beginning.

The analysis of the material removed must be coherent with the melting temperature of each material. Therefore, the displayed melting temperatures when measuring the material removed must match the melting temperature of the steel and the aluminium. In the tool, that temperature equals 1535 °C and matches the border between the grey and the red area, when the temperature of the simulation is analysed, with a maximum of 1535 °C. On the other hand, when the workpiece analysis is done, that temperature equals 565 °C and matches the end of the coloured area, with a minimum of 565 °C.

**Figure 7:**
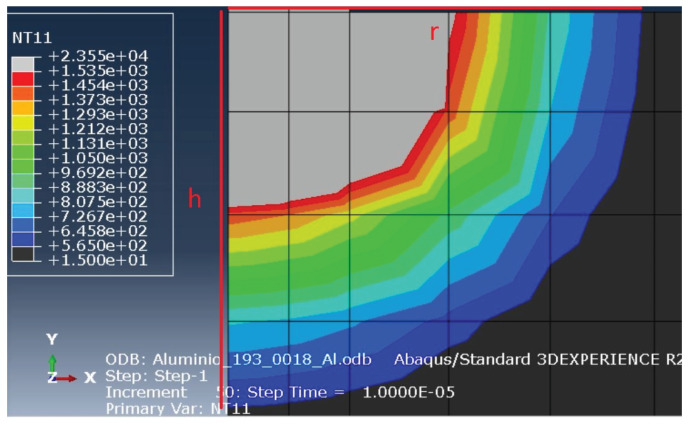
Measurement of the removed volume from the workpiece.

**Figure 8:**
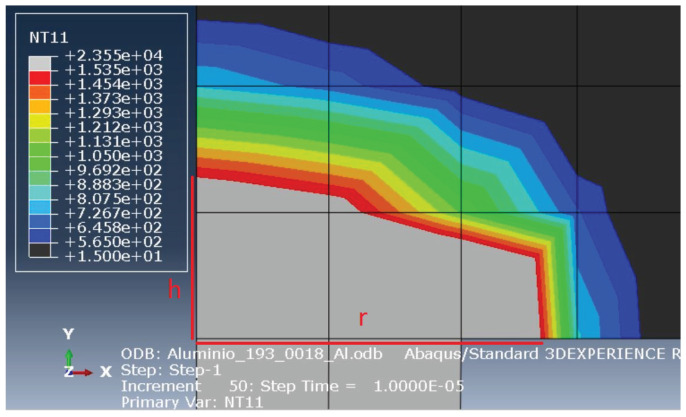
Measurement of the removed volume from the tool.

To obtain the amount of removed material from the tool, the radius of the molten area and its height were measured from the beginning of the discharge channel up to the limit of the grey area, horizontally (*r*) and vertically (*h*). On the other hand, to obtain the volume of removed material from the workpiece, the same procedure was performed, but limited by the end of the coloured area. This method is shown in Figure 7 and Figure 8. Equation (16) was then applied to calculate the volume. The numerical value of the surface roughness, *R*z, is equal to the value correspondent to the depth of the crater, *h*.

In order to calculate the performance parameters, the erosion time considered was the sum of the time-on and time-off and the volume of removed material was calculated as shown previously. As for the material removal rate, it can be calculated by dividing the volume of material removed from the workpiece by the erosion time.

(17) $$MRR=\frac{Volume}{Erosion\;Time}$$

The tool wear rate can be calculated by dividing the volume of material removed from the tool by the erosion time.

(18) $$TW=\frac{Volume}{Erosion\;Time}$$

Finally, in order to obtain the tool wear ratio, one must divide the TW by the MRR.

(19) $$TWR=\frac{TW}{MRR}$$

The results are shown in Table 3.

### 3.2. Experimental Results

In each of the experiments, both the electrode and the workpiece were weighed three times before and after the EDM process, in order to calculate the volume reduction through their densities. The erosion time was also recorded so that the basic parameters could be calculated. The results obtained in this experiment are shown in Table 4.

The numerical values of the surface roughness can be seen in Table 5. The *R*max represents the maximum values of the average maximum surface height of the workpiece, *R*z.

## 4. Discussion

**Figure 9:**
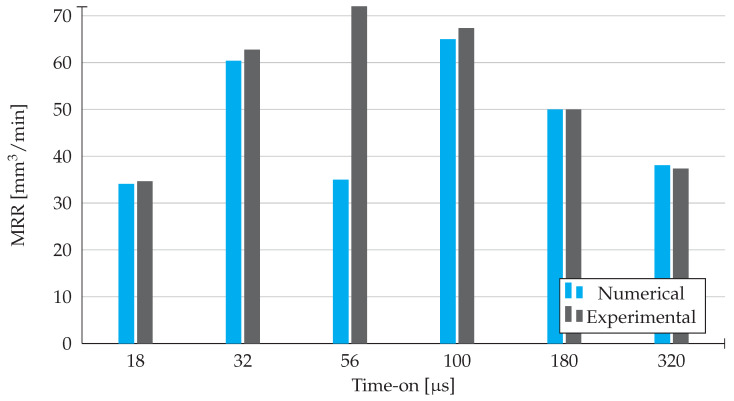
Material Removal Rate results obtained from experimental work and numerical simulation in Abaqus.

As it is shown in Figure 9, the material removal rate has a tendency to increase up to 56 μs, where it reaches a maximum and then drops for longer time-on durations. This tendency is visible for the experimental results. However, with the exception of the 56 μs value, the numerical values respect the exact same pattern. This specific inconsistency is justified by the possible variations in time-on, which are a consequence of the inability to minutely control these same fluctuations for higher time-on values. Consequently, we obtain a certain consistency with the theoretical research done. In conclusion, the higher values of material removal rate are obtained for intermediate values of time-on.

In addition, we can see that there are certain variations between the numerical and experimental results about MRR, where for the time-on values of 18, 32, 56, 100, and 180 μs the experimental values are higher and for the time-on value of 320 μs the numerical result is higher. These variations are due to the hydraulic forces that are a result of the electrical potential at the end of the discharge, along with the debris on the surface that create some irregularities on the surface. As the numerical results do not take into account these forces, there can be some positive or negative variations in the amount of removed material between the experimental and numerical values. Additionally, the fact that the numerical results are based on the fact that the amount of removed material is considered equivalent to a perfect spherical cap (by using Equation (16)) may create some disparities between the simulation results and the reality. This aspect has an impact in both the tool and the workpiece, influencing the MRR, TW and TWR values.

For all the values of time-on analysed, the results obtained in the simulation and experimental work were significantly similar with little relative error. If we disregard the exception made previously, the highest disparity we can find here is for 32 μs, where the relative error of the numerical results is roughly 3.8%. In conclusion, the approximation of the results is very reliable.

**Figure 10:**
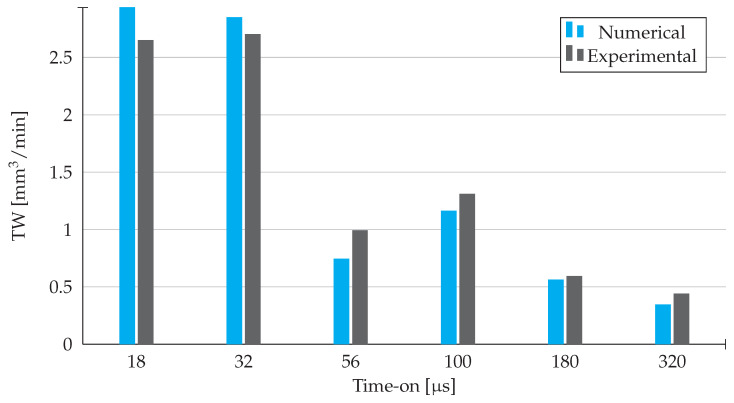
Tool Wear Rate results obtained from experimental work and numerical simulation in Abaqus.

By analysing the Figure 10, two distinct ranges of values can be observed: one for shorter time-on values, namely for 18 and 32 μs, and another for the higher values. Additionally, for both the numerical and experimental results, we can see a drop of tool wear rate values with the increase in the time-on implemented. However, there is a slight increase in tool wear rate for 100 μs, noticeable on the results obtained by both methods. Nonetheless, for the remaining values of time-on, the acquired results are frankly similar, once again.

Conclusively, the minimum tool wear rate outputs come for longer time-on values, since the amount of volume removed from the used tool is heavily reduced.

**Figure 11:**
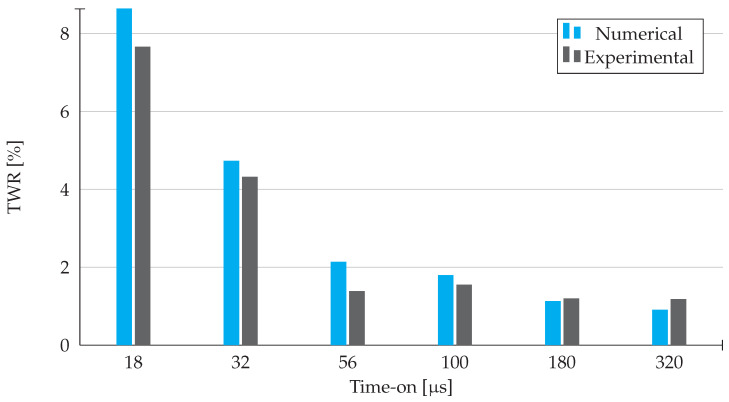
Tool Wear Ratio results obtained from experimental work and numerical simulation in Abaqus.

As it is possible to see in Figure 11, the drop of the tool wear ratio values is accompanied by the increase in the respective time-on values for both the experimental and numerical results. However, the experimental results for 56 μs slightly break the pattern, since the slight decrease that occurred on the tool wear rate values will mathematically influence the final value of tool wear ratio for this specific time-on value.

As it was verified in both the MRR and TW, there is a reasonable approximation between the simulation and the experimental results, apart from the results obtained for 56 μs, that are a consequence of the mathematical propagation of the relative error obtained on the material removal rate results.

In conclusion, the best TWR values are for higher time-on values, since the electrode efficiency is higher.

**Figure 12:**
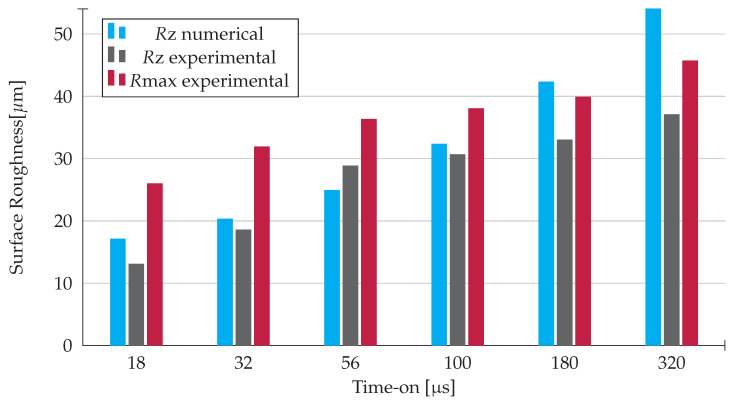
Surface roughness of the workpiece as a function of time-on.

As it can be seen in Figure 12, there is an increase in the surface roughness with the increase in the time-on duration. It was expected that the surface roughness of the workpiece would increase with the volume of material removed and this prediction was confirmed by both the numerical and experimental results.

For each time-on, there is a slight difference between the numerical and experimental *R*z, which might be due to the change in fusion temperature of the workpiece material, consequence of the increase in pressure that is not accounted for in the numerical model.

This already mentioned difference is more evident in the time-on durations of 180 and 320 μs. As already stated, this disparity is a consequence of the existing variations in time-on, which derive from the difficulty to control these same fluctuations for higher time-on values with precision.

## 5. Conclusions

The present thermal–electrical model using a trunk-conical discharge channel to simulate the electrical discharge machining phenomenon leads to accurate results when compared to the experimental results.

The present thermal–electrical model is an integrated model where the distribution of energy is done taking into account the boundary conditions, mainly the applied voltage that affects the position of the heat source relatively to both electrodes, the tool and the workpiece.

The results are obtained with a pair of electrodes never used by other researchers, the mild steel tool and aluminium workpiece, instead of the traditional copper tool and aluminium workpiece. The results of the present research show that the steel can be the tool material when working aluminium because the output parameters show trends very similar to other pairs of electrode materials. The present model allow the users to choose a different tool material for a chosen workpiece.

The parameter that best reflects the data treatment performed and, therefore, the yield of the EDM process, is the tool wear ratio, since the purpose of the EDM is to remove the largest amount of material in the part causing the least possible wear on the tool used. We can conclude that it is for longer impulse values that the tool wear ratio percentages are lower and, consequently, more favourable. However, considering different applications, the surface roughness cannot be disregarded and its influence ought to be taken into account.

If the purpose of EDM is to remove the most volume of material in the shortest time, one should aim for the highest material removal rate. Nevertheless, the larger the workpiece’s removed volume is, the higher the surface roughness values will be. If having the highest electrode efficiency is the goal, one should aim for the lowest tool wear ratio. On the other hand, if a low surface roughness is of extreme importance, this can be achieved with lower time-on durations, although the efficiency of the electrode might be compromised.

Consequently, the steel–aluminium pair of electrodes showed a performance behaviour similar to the copper–steel pair of electrodes, where the steel is, in most cases, the workpiece and, in this specific case, the tool.

In all of the electrode performance parameters, there is a fairly good approximation between the numerical and the experimental results.

The discrepancy that occurs for the discharge duration of 56 μs is explained by its numerical surface roughness compared to all the other values that lead to accurate approximations between numerical and experimental results. Therefore, the numerical surface roughness value, obtained for 56 μs, is the only one that is inferior to the experimental surface roughness, when it should present a similar value to the numerical surface roughness relative to the pulse duration of 100 μs. Consequently, this means that the applied energy value is less than the experimental, inducing a lower material removal rate and a higher tool wear rate, which globally implies a higher tool wear ratio.

To conclude, this model is a considerably good approximation of the EDM process, even though there should be a continuous improvement and development of this intriguing process, as the obtained results seriously converge more and more to the experimental data obtained. This means we are getting closer to a precise model of simulation that can recreate the EDM process with minimal error.

## Figures and Tables

**Table 1:** Material properties [7].

Property	Channel	DIN 3.4365	DIN CK45
Thermal conductivity [W/cm·K]	1.6	1.3 @ 25 °C	0.58 @ 25 °C
		1.42 @ 100 °C	0.49 @ 300 °C
		1.76 @ 500 °C	0.38 @ 600 °C
Density [kg/cm${}^{3}$]	0.00027	0.0028	0.007849
Electrical conductivity [(Ohm·cm)${}^{-1}$]	75,000	194,170	56,500
Joule heat fraction	1	1	1
Specific heat [J/kg·K]	7440	960	486

**Table 2:** Experimental parameters.

Parameter	Numerical Value
Pulse Intensity	19.3 A
Time-on	18, 32, 56, 100, 180, 320 μs
Time-off	18, 32, 56, 100, 180, 320 μs
Electric Potential	180 V
M/2Gap	0.7/0.36 mm

**Table 3:** Numerical results.

Time-On [μs]	MRR [mm${}^{3}$/min]	TW [mm${}^{3}$/min]	TWR [%]
18	34.0	2.938	8.64
32	60.3	2.848	4.72
56	34.9	0.742	2.13
100	64.9	1.160	1.79
180	49.9	0.560	1.12
320	38.0	0.343	0.90

**Table 4:** Experimental results.

Time-On [μs]	MRR [mm${}^{3}$/min]	TW [mm${}^{3}$/min]	TWR [%]
18	34.6	2.648	7.65
32	62.7	2.701	4.31
56	72.0	0.991	1.38
100	67.3	1.037	1.54
180	49.9	0.592	1.19
320	37.3	0.438	1.17

**Table 5:** Numerical values of the surface roughness as a function of time-on.

Time-On [μs]	*R*z Experimental [μm]	*R*z Numerical [μm]	*R*max [μm]
18	13.07	17.1	25.97
32	18.53	20.3	31.87
56	28.81	24.9	36.31
100	30.64	32.3	38.03
180	33	42.3	39.88
320	37.05	54.1	45.67

## Data Availability

Data is contained within the article.

## Abbreviations

The following abbreviations are used in this manuscript:

AC	Adaptive Control
ACO	Adaptive Control Optimisation
EDM	Electrical Discharge Machining
FE	Finite Element
FEA	Finite Element Analysis
FEM	Finite Element Method
HAZ	Heat Affected Zone
MRR	Material Removal Rate
TW	Tool Wear Rate
TWR	Tool Wear Ratio
